# Feasibility and safety of fine positioning needle-mediated breathing control in CT-guided percutaneous puncture of small lung/liver nodules adjacent to diaphragm

**DOI:** 10.1038/s41598-021-83036-z

**Published:** 2021-02-09

**Authors:** Qingde Wu, Bihui Cao, Yujin Zheng, Baoxia Liang, Manting Liu, Lu Wang, Jinling Zhang, Liyan Meng, Shaoyong Luo, Xuxia He, Zhenfeng Zhang

**Affiliations:** 1 Department of Radiology, Shunde Chinese Medicine Hospital, the Affiliated Hospital of Traditional Chinese Medicine University of Guangzhou, Foshan, 528333 China; 2 Department of Radiology, The Second Affiliated Hospital of Guangzhou Medical University, Guangzhou, 510260 China

**Keywords:** Cancer, Medical research, Oncology

## Abstract

To assess the efficacy, safety, and feasibility of a separate inserted positioning fine needle-mediated breathing-control technique applied to computed tomography (CT)-guided percutaneous puncture for biopsy or microwave ablation (MWA) of small lung/liver nodules near diaphragm. Total 46 patients with pulmonary/liver small nodules (≤ 3 cm in size) near diaphragm(nodule within 1 cm distance to the diaphragm)were undergone percutaneous biopsy ( n = 15) or MWA (n = 31) under the guidance of CT, and a separate positioning fine needle-mediated breathing-control technique was applied for the precise punctures. CT plain scan was performed to monitor the complications after the procedure. The patient baseline data, operation details, successful rate, major complications as well as radiation dose during the procedure were recorded and analyzed. With the assistance of a fine positioning needle insertion for controlling the breathing, the puncture success rate for biopsy or MWA reached 91.30% (42/46). For biopsy, the mean nodule diameter, nodule distance to the diaphragm, puncture time and radiation dose during CT scan were 2.27 cm ± 0.74, 0.61 cm ± 0.24, 18.67 min ± 6.23, 28.84 mSv ± 6.99, respectively; For MWA, the mean nodule diameter, nodule distance to the diaphragm, puncture time and CT radiation dose were 2.35 cm ± 0.64, 0.69 cm ± 0.23, 38.71 min ± 13.65, 33.02 mSv ± 8.77, respectively. Totally, there were three and four cases found minimal puncture-related hemoptysis and pneumothorax needed no additional treatments, respectively. We recently developed and verified a feasible, safe and highly effective puncture technique with reasonable radiation dose for CT-guided biopsy or MWA for small nodules abutting diaphragm, therefore worthy of extensive application to similar clinical situations.

## Introduction

Cancer is one of the major public health concerns worldwide, especially the lung and liver cancers, which are the leading causes of cancer related death in China^1,2^. In the era of personalized precision medicine, obtaining an accurate biopsy histopathological diagnosis or performing suitable local treatment such as ablation is critical for patients with advanced malignance^3,4^.

Published papers have demonstrated high accuracy and reliability for percutaneous lung nodule biopsy under the guidance of CT imaging. Recently, a CT-guided single-center study with 750 biopsies of pulmonary nodules revealed high diagnostic accuracy and acceptable complication rates^5^. However, small pulmonary nodules near the diaphragm are still challenging to be targeted effectively and safely for biopsy or ablation due to obvious influence by respiratory motion^6^.

Similarly, to puncture a small liver nodule abutting a diaphragm dome for biopsy or ablation also remains a critical issue, though several approaches, such as artificial ascites, laparoscopy-guided transthoracic trans-diaphragmatic, as well as CT- or ultrasound-guidance have been addressed^7–10^.

In order to improve the puncture success rate for those small nodules near the diaphragm dome, we have recently developed a novel but feasible technique by applying a separate positioning percutaneous fine-needle (≥ 23G) to indicate and control the breathing for the next percutaneous biopsy or ablation puncture. Here, we retrospectively summarized all puncture cases with lung or liver nodules in size of 3 cm or less and within only 1 cm distance to the diaphragm in our department during past 3–4 years. The puncture performance, complications and procedure related details by this new technique were evaluated and analyzed.

## Materials and methods

### Patients

**Figure 1:**
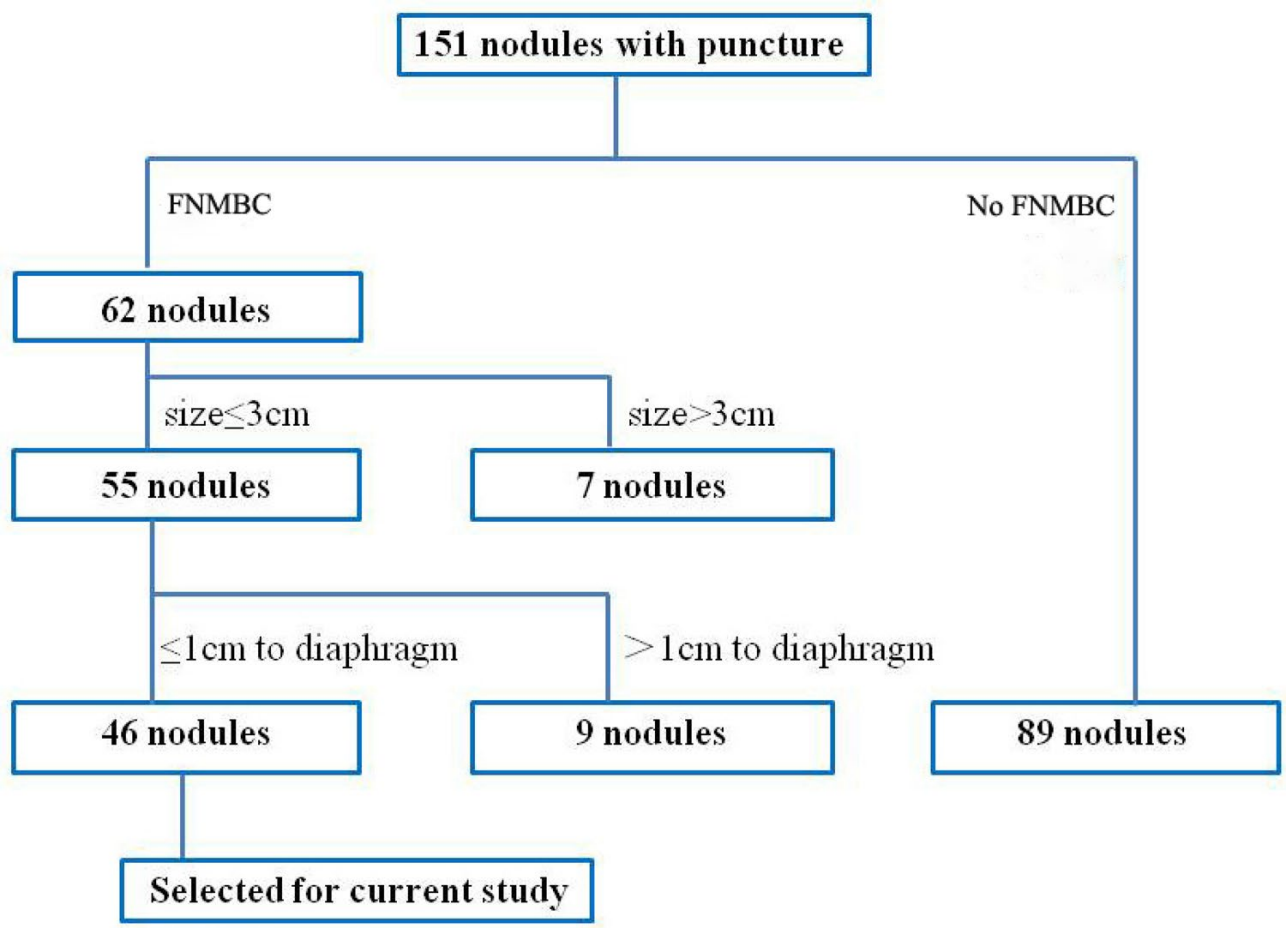
Flow chart of patient selection for this study. FNMBC: Fine needle-mediated breathing control.

We retrospectively collected 151 patients underwent conventional CT (BrightSpeed Series CT systems, GE Healthcare, Milwaukee, WI, USA) guided percutaneous biopsy or microwave ablation for lung or liver nodules near the diaphragm between Jan. 2016 and Dec. 2017 at our department, and 46 consecutive patients (46 nodules, all ≤ 3 cm) were enrolled in this study. The nodules were considered abutting to diaphragm if the distance between diaphragm and closest tumor edge was ≤ 1 cm on chest or abdominal CT images evaluated in the coronal or sagittal image before puncture^11^. Those with history of encephalopathy, severe respiratory disease that needed respirator or myocardial infarction, portal thrombosis, Child–Pugh Class C or D, ascites refractory to diuretics, variceal bleeding that would influence the procedure were excluded. In case of puncture bleeding, platelet count > 50,000 cells/mm^3^ is required, coagulation parameters (e.g., prothrombin time or activated prothrombin time) were confirmed to ensure that patient had taken no anticoagulants within 3 days before the procedure. The decision whether to perform biopsy or ablation was routine determined by a multidisciplinary team (MDT) of physicians coming from different departments. The use of a separate fine-needle insertion to mediate biopsy or ablation puncture of the nodules was approved by our institutional review board (IRB) in Shunde Chinese Medicine Hospital. Case selection details are shown in Fig. 1.

Of 46 patients, there were 15 for biopsy (15 nodules, 5 in lung, 10 in liver) and 31 for MWA (31 nodules, 7 in lung, 24 in liver). There were 4 females and 11 males involved in biopsy, the mean age, nodule diameter, nodule distance to the diaphragm, prothrombin time were 60.53 year ± 12.39, 2.27 cm ± 0.74, 0.61 cm ± 0.24, 12.85 s ± 0.89, respectively. For MWA, there were 7 female and 24 male patients, the mean age, nodule diameter, nodule distance to the diaphragm, prothrombin time were 56.19 year ± 10.04, 2.35 cm ± 0.64, 0.69 cm ± 0.23, 12.94 s ± 1.51, respectively. There were 4 biopsy and 11 MWA patients who had history of cancers listed in Table1. For other 31 patients (11 biopsy, 20 MWA), routine triphase CT for liver nodules and contrast CT for lung nodule were performed to identify the features of them, all the diagnosis results were highly suspected cancer which need further detect or therapy. For all MWA, either a quick-frozen biopsy was obtained to ensure malignancy or clinical diagnosis of metastasis was made by a MDT group.

**Table1:** Patients baseline.

Characteristics	Biopsy (15)	MWA (31)
**Sex**		
Female	4	7
Male	11	24
**Age (years)**		
Range	41–79	39–80
Mean ± *SD*	60.53 ± 12.39	56.19 ± 10.04
**Nodule location**		
Lung	5	6
Liver	10	25
Nodule size(cm)	2.27 cm ± 0.74	2.35 cm ± 0.64
Distance to diaphragm(cm)	0.61 cm ± 0.24	0.69 cm ± 0.23
Prothrombin time (PT, s)	12.85 s ± 0.89	12.94 s ± 1.51
**History of tumors**		
Intestinal cancer	1 ( P 0, L 1)	4 ( P 0, L 4)
NPC	2 ( P 0, L 2)	3 ( P 2, L 1)
Esophageal carcinoma	0	2 ( P 1, L 1)
Cervical cancer	0	1 ( P 1, L 0)
Renal carcinoma	0	1 ( P 1, L 0)
Breast cancer	1 ( P 0, L 1)	0

SD, NPC, P, L and MWA is abbreviation of standard deviation, nasopharyngeal carcinoma, pulmonary, liver and microwave ablation respectively.

### CT scanner

A multi-detector helical CT scanner (Bright-Speed Series CT systems, GE Healthcare, Milwaukee, WI, USA) was used, with the simultaneous acquisition of 16 slices per full rotation. The technical parameters were: 120 kV, 200–350 mAs, slice 5 mm, reconstruction increment 5 mm. Scanned regions were determined according to the nodule location, and a small range covering the target nodule to minimize the scan-related radiation dose was considered. After each scan, the radiation dose and puncture time were recorded.

### Needles

Fine needle: A 15 cm long, 23Gauge fine needle (Angiotech, Vancouver, CA.) was inserted near the nodule in the lung used as respiratory marker before biopsy or ablation needle puncture during the procedure.

Biopsy needle: A coaxial biopsy needle device (10-15 cm long, 14-18Gauge in diameter; Angiotech, Vancouver, CA) was applied to obtain tissue samples.

Ablation device: An ablation system (Nanjing ECO Medical Equipment Co., Ltd., Nanjing, China ) equipped with 10 cm, 15 cm, or 20 cm ablation electrode with 19Gauge in diameter was employed in our clinical practice. The appropriate power and time were determined by tumor size and location.

### Procedure

Prior to puncture, all nodules were routinely CT scanned to determine the appropriate operation position (supine, prone or lateral). The risks and benefits were explained to the patient before the punctures and informed consent was obtained from each patient before the procedure.

**Figure 2:**
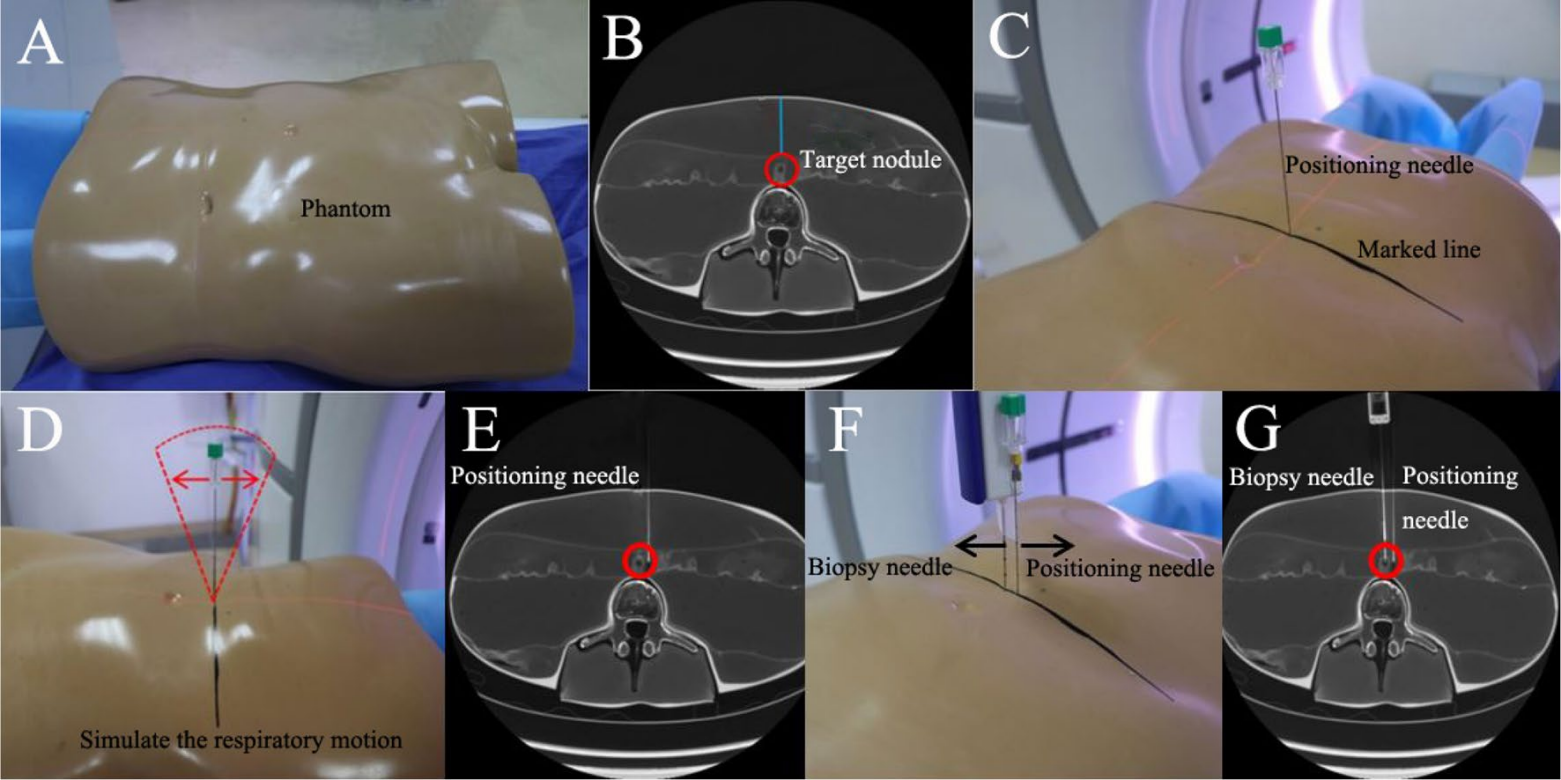
Schematic of a fine needle-mediated breathing control puncture for biopsy, illustrated by a body phantom (**A**). (**B**) The phantom was CT scanned to show the target nodule (red circle) and puncture path (green line); (**C**) a 23G fine needle was vertically inserted with the help of laser light, and the project line of fine needle delineated (dark line); (**D**) The fine needle was simulating swinging with the breathing and was inserted in the vertical position; (**E**) the fine needle near the target nodule was showed in one transverse CT image to confirm the fine needle was seen in one slice image; (**F**) With the guidance of the fine needle, a biopsy needle was punctured; (**G**) both needles were verified by CT scan.

**Figure 3:**
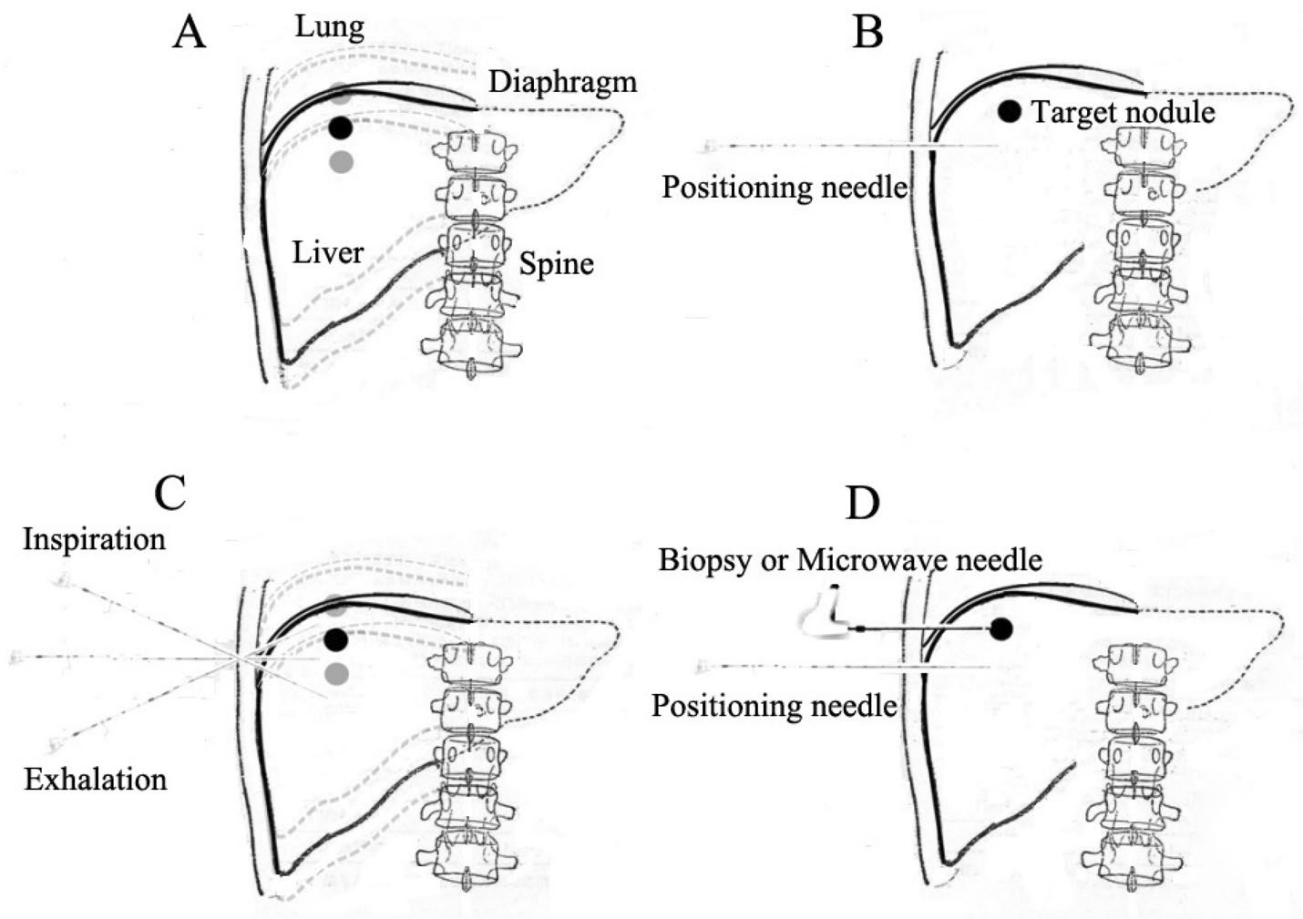
Illustrating picture of a fine needle-mediated breathing control for biopsy/MWA puncture technique for a liver nodule near diaphragm. (**A**) A nodule was showed in liver with 1 cm distance from its up edge to diaphragm swinging up and down with respiratory movement; (**B**) Patient was trained to hold his breath (no matter expire or inspire), a 23G fine needle was inserted near the target nodule (usually 1-5 cm away from the nodule) to serve as a breathing indicator; (**C**) The fine needle acted as a breathing marker. (**D**) While the fine needle was visible on one CT transverse image (right on vertical position to the skin), a biopsy/ablation needle was pierced into the liver nodule as planned and confirmed by CT scan.

**Figure 4:**
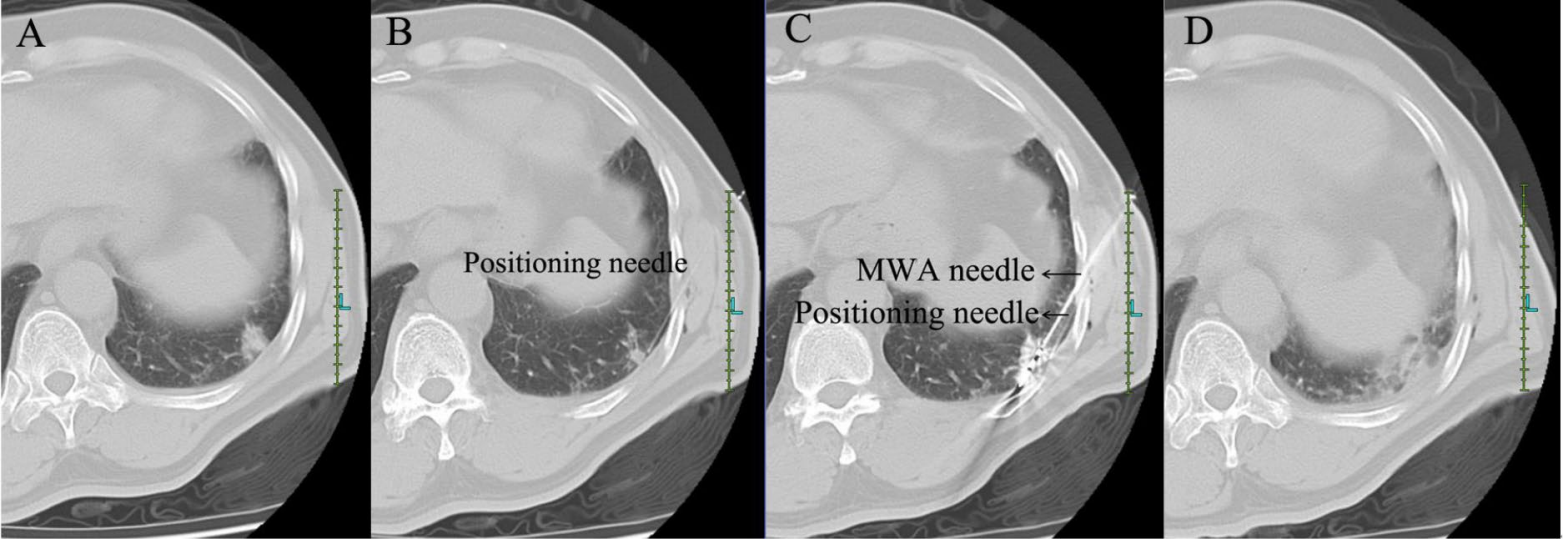
A clinical MWA puncture case by applying a fine needle-mediated breathing control technique for a 59-year old male who had esophageal carcinoma history. (**A**) Routine CT scan showed the feature of the lesion; 1.7 cm × 1.6 cm × 2.0 cm in size and located at posterior basal segment of lower lobe of the left lung. (**B**) A 23G fine needle was inserted into the lung as breathing indicator, showing the needle paralleled to the CT cross section and the whole length of needle visible in the image. (**C**) With the reference of the fine needle, a MWA electrode was inserted to the distal margin of the lesion. (**D**) Post-ablation CT scan observed no obvious complications.

The puncture details were as follows: (a) A planning CT scan was performed to design a proper piercing path from puncture point to target nodule; the projected laser line(on the body)was drew to be a position marker (black) of a fine needle (Fig. 2A–C); (b) At the skin puncture point, 3-10 ml of 1% lidocaine hydrochloride was administrated subcutaneously as local anesthesia for biopsy. For MWA, general anesthesia was required. (c) Patients were trained to hold his/her breath (no matter expire or inspire), a 23G fine needle was vertically inserted near (3-5 cm away from) the planned puncture path (Figs. 2C, 3B). Here, fine needle (usually on the marked black line) was serviced as a real-time breathing indicator to maintain the same respiration phase ensuring the fine needle in right vertical position while a second puncturing for biopsy or ablation. (d) At the same respiration phase with fine needle in vertical position, patient was asked to hold her/his breath to complete CT scan to check whether the fine needle is viewable on one CT transverse image only, indicating that the needle was paralleled to the emission direction of X-ray (Phantom) (Fig. 2E). (e) A coaxial biopsy needle or MWA electrode was inserted into proximal margin (biopsy) or distal margin (MWA) of nodule (Figs. 2F, 3D), and the biopsy/ MWA needle reached the target nodule ensured by further CT scan (Fig. 2G). (f) After the procedure, chest radiographs or CT plain scan was routinely scheduled to detect pneumothorax, intrapulmonary hemorrhage, phrenic nerve injury, pleural effusion, ascites, subcapsular hematoma, or diaphragmatic hernia. Figures 2 and 3 are brief illustrations showing the puncture procedure. Figure 4 is a representative clinical case applied with the fine needle-mediated breathing-control ablation technique.

### CT scan radiation dose assessment

The CT scan effective dose (ED) can be estimated by multiplying the scanned DLP and a conversion factor also named k value. The ED is related to the scan region, which directly corresponds to different organs with different tissue weighting factors^12^. Here, k value of 0.015 mSv/mGy-cm used in this study is consistent with Bongartz et al.^13^. The effective dose for each procedure (targeting, monitoring, and post-ablation survey) was calculated by summing the effective dose for each CT scan.

### Statistical analysis

Data analysis was calculated via using Excel 2007 (Microsoft, Redmond, WA). Procedure details, complication rate as well as effective radiation dose were statistically evaluated. Continuous data were expressed as mean ± deviation. Categorical data were expressed by count with percentage.

## Results

### Biopsy or MWA data

In this study, technical success for biopsy was defined as obtained adequate material for pathologic analysis; for MWA, technical success was defined as right targeting the nodule, which guaranteed the following successful procedure. Besides one biopsy patient and three MWA patients did not achieve technique success, the total technical success was achieved in 42 of the 46 procedures (91.30%). All the biopsy and 29 MWA patients were punctured by supine position, others by prone position. All punctures were performed under the guidance of the fine positioning needle. The mean procedure time were 18.67 min ± 6.23 (biopsy) and 38.71 min ± 13.65 (MWA). The mean puncture depth was 5.30 cm ± 1.27 (biopsy) or 10.52 cm ± 2.97 (MWA) (Table 2).

**Table 2:** Puncture-related data.

Characteristics	Biopsy (15)	MWA (31)
Technical success	93.33%	90.32%
Puncture time (min)	18.67 min ± 6.23	38.71 min ± 13.65
Patient position		
Supine	15	29
Prone	0	2
Pneumothorax	0	4
Hemoptysis	2	1
Depth of puncture (cm)	5.30 cm ± 1.27	10.52 cm ± 2.97
CT scan number	1.33 ± 0.62	3.23 ± 1.09

1. The data were presented with mean ± Standard Deviation or cases. 2. MWA, microwave ablation.

Lung nodule biopsy pathology results showed four lung adenocarcinoma and one squamous cell carcinoma. Liver nodule biopsy pathology results showed four primary hepatocellular carcinoma, one cholangiocarcinoma, four cases of metastases from NPC (2 cases), intestinal cancer (1 case), or breast cancer (1 case), respectively; and one case of inflammation as well (Table 3).

**Table 3:** Biopsy pathology result of nodules.

Characteristics	Result
Lung	5
Adenocarcinoma	4
Squamous cell carcinoma	1
Liver	10
Hepatocellular carcinoma	4
Cholangiocarcinoma	1
NPC metastasis	2
Intestinal cancer metastasis	1
Breast cancer metastasis	1
vInflammation	1

NPC, nasopharyngeal carcinoma.

### Complications

About puncture-related complications, only three cases (two biopsy, one MWA) found mild hemoptysis (self-limited), and four cases (MWA) had mild pneumothorax (< 5%) needed no further management and following up CT showed pneumothorax absorbance within one week after the procedure. No delayed pneumothorax or puncture related death occurred. No phrenic nerve injury, pleural effusion, ascites, subcapsular hematoma, or diaphragmatic hernia was found in these patients (Table 2).

### Radiation dose

In this study, the puncture-related X-ray radiation dose is our major consideration since our aim was to propose a novel puncture technique to largely improve the puncture efficacy with minimal radiations. For biopsy, effective dose (ED, mean ± standard deviation) of planning scan, fine needle inserting, and biopsy needle inserting and post-biopsy scan were calculated as 8.57 mSv ± 2.42, 3.15 mSv ± 0.80, 7.67 mSv ± 5.76, 9.45 mSv ± 2.42, respectively. For MWA, the mean effective doses of planning, fine needle inserting, MWA needle inserting, ablation monitoring, and post-ablation scanning were 6.66 mSv ± 2.61, 3.61 mSv ± 1.27, 9.78 mSv ± 5.13, 4.34 mSv ± 2.41, 8.63 mSv ± 2.40, respectively (Table 4).

**Table 4:** Effective radiation dose.

Phases	Biopsy		MWA	
	DLP(mGy*cm)	ED(mSv)	DLP(mGy*cm)	ED(mSv)
Before biopsy/MWA				
Planning CT	571.33 ± 161.41	8.57 ± 2.42	444.01 ± 173.91	6.66 ± 2.61
Fine needle	210.13 ± 53.65	3.15 ± 0.80	240.49 ± 84.42	3.61 ± 1.27
Biopsy/MWA needle	511.03 ± 383.79	7.67 ± 5.76	651.93 ± 342.00	9.78 ± 5.13
MWA monitoring	NA	289.49 ± 160.79	4.34 ± 2.41	
Post biopsy/MWA survey	630.22 ± 161.15	9.45 ± 2.42	575.41 ± 160.23	8.63 ± 2.40
Total procedure	1922.71 ± 465.96	28.84 ± 6.99	2201.33 ± 584.77	33.02 ± 8.77

DLP, ED, MWA, are the abbreviations of dose length product, effective dose, and microwave ablation, respectively.

## Discussion

In current study, we showed a feasible, safe, and novel percutaneous biopsy or ablation technique with a positioning fine needle guidance under CT with a puncture success rate reached 91.30%. In addition, the total effective radiation dose of biopsy and MWA is only 28.84 mSv ± 6.99 and 33.02 mSv ± 8.77, respectively. These results indicated that with the application of a separate fine needle while puncturing for those small lung/liver nodules near the diaphragm, we could completely control the respiration phase of the patient to eliminate influence of respiratory movements, so as leading to an improved puncture success rate and a relative reasonable radiation exposure.

Reported studies demonstrated that the technique success rate was significantly lower, whereas the complications were higher for lung/liver smaller nodules abutting the diaphragm to puncture ^11,14^. The existing difficulty to puncture a small size pulmonary lesion and its low success rate might due to the small size itself and respiratory movement, because a slight error of piercing angle would render the needle miss the target^15–17^. What is more, repeated multiple punctures could result in more injury of pleural and lung tissue^15–18^. Therefore, the puncture success rate for these nodules near the diaphragm is relatively lower and the complications may relatively higher, when compared to these liver/lung nodules far from the diaphragm^11,14^. However, in our study, with the help of a positioning fine needle, a higher puncture success rate (91.30%) and a lower complication rate (8.7% for pneumothorax, 6.52% for hemoptysis) were achieved compared to those previous studies^16,19,20^. This method overall has several advantages: (1) the fine needle could anchor the tissue around the nodule, rendering the needle, nodule, chest wall and diaphragm move as a whole while breathing; (2) the swing of the positioning needle can help determine whether the patient really holds his/her breath, which is helpful to reduce the injury of the pleura when puncturing.

A higher radiation dose potentially means a higher risk of cancer^12^. However, few published studies paid attention to radiation dose of patients undergoing CT interventional procedures such as percutaneous biopsy or ablation. Tsalafoutas et al.^21^ concluded that the effective radiation dose of CT-Guided biopsy and radiofrequency ablation were 23 mSv and 35.3 mSv, respectively, and also demonstrated that the largest contribution of effective dose was needle targeting. Park et al.^22^ suggested in their study that the total estimated effective dose of CT-guided percutaneous cryoablation of liver tumors is 72 ± 18 mSv, and indicated that the effective dose of targeting dose (37.5 ± 12.5 mSv, 24.20 ± 8.13 mSv in our study) was the largest component compared to the effective dose of the planning phase (4.8 ± 2.2 mSv), monitoring phase (25.5 ± 6.8 mSv), and post-ablation survey (4.1 ± 1.9 mSv) phase during the procedure. Interestingly, our evaluated total effective dose (28.84 ± 6.99 for biopsy, 33.02 ± 8.77 mSv for MWA) is comparable to those of Tsalafoutas et al.^21^. and dramatically lower than that of Park et al.^22^. In our study, a fine needle was used and it seemed that extra CT scan is needed and thus more radiation dose produced, however, it was the needle that controlled the breathing, facilitated the puncture procedure, and reduced the radiation dose consequently.

According to our clinical experience, the breath-holding training of patient before the operation is required, especially for the elderly and "move to scan" button should be prepared timely before the CT scan. To control the total breath-holding time less than 20 s is suggested.

There are limitations in this study. First, our study had a retrospective design which may introduce bias. Second, the small population was derived from a single institution and the long-term follow-up period was absent. Third, our study design did not include a comparison with the conventional puncture method. Thus, we could not directly evaluate the advantages brought by the fine needle mediated breathing control method. However, our major aim here is to assess the feasibility and safety of the fine needle mediated breathing control method, further prospective studies are necessary to warrant its efficacy in the future.

In conclusion, the novel puncture technique applied in current study demonstrated that a fine needle mediated breathing control method could eliminate influence of respiratory movement effectively, help to guide precision puncture of hard-reachable small nodules in lung and liver, and yield to reduced complications and radiation dose for patients, which might have significant clinical value in routine practice.

### Ethical approval

All procedures performed in studies involving human participants were in accordance with the ethical standards of the institutional and/or national research committee and with the 1964 Helsinki Declaration and its later amendments or comparable ethical standards.

### Informed consent

For this type of study, formal consent is not required.

## Abbreviations

CT: Computed tomography
MWA: Microwave ablation
IRB: Institutional review board
G: Gauge
ED: Effective dose
DLP: Dose length product
NPC: Nasopharyngeal carcinoma
MRI: Magnetic resonance imaging
RFA: Radiofrequency ablation
